# The role of profilin-1 in endothelial cell injury induced by advanced glycation end products (AGEs)

**DOI:** 10.1186/1475-2840-12-141

**Published:** 2013-10-04

**Authors:** Zhenyu Li, Qiaoqing Zhong, Tianlun Yang, Xiumei Xie, Meifang Chen

**Affiliations:** 1 Department of Geriatric Medicine, Xiang-Ya Hospital, Central South University, Xiang-Ya Road 87#, Changsha, Hunan 410008, China; 2 Department of Cardiology, the First People’s Hospital of Chenzhou, Chenzhou, Hunan 423000, China; 3 Department of Cardiology, Xiang-Ya Hospital, Central South University, Changsha, Hunan 410008, China

**Keywords:** Advanced glycation end products (AGEs), Profilin-1, Endothelial cells, Cytoskeletal rearrangement, Reactive oxygen species (ROS), Nuclear factor kappa B (NF-κB), Protein kinase C (PKC)

## Abstract

**Background:**

Accumulation of advanced glycation end products (AGEs) in the vasculature triggers a series of morphological and functional changes contributing to endothelial hyperpermeability. The reorganisation and redistribution of the cytoskeleton regulated by profilin-1 mediates endothelial cell contraction, which results in vascular hyperpermeability. This study aimed to investigate the pivotal role of profilin-1 in the process of endothelial cell damage induced by AGEs.

**Methods:**

Human umbilical vein endothelial cells (HUVECs) were incubated with AGEs. The mRNA and protein expression of profilin-1 was determined using real-time PCR and western blotting analyses. The levels of intercellular adhesion molecule-1 (ICAM-1), nitric oxide (NO) and reactive oxygen species (ROS), as well as the activities of nuclear factor-κB (NF-κB) and protein kinase C (PKC), were detected using the appropriate kits. The levels of asymmetric dimethylarginine (ADMA) were determined using HPLC. The distribution of the cytoskeleton was visualised using immunofluorescent staining.

**Results:**

Compared with the control, incubation of endothelial cells with AGEs (200 μg/ml) for 4 or 24 h significantly up-regulated the mRNA and protein expression of profilin-1, markedly increased the levels of ICAM-1 and ADMA and decreased the production of NO (*P*<0.05, *P*<0.01), which was significantly attenuated by pretreatment with DPI (an antioxidant), GF 109203X (PKC inhibitor) or BAY-117082 (NF-κB inhibitor). DPI (10 μmol/L) markedly decreased the elevated levels of ROS induced by AGEs (200 μg/ml, 24 h); however, GF 109203X (10 μmol/L) and BAY-117082 (5 μmol/L) exhibited no significant effect on the formation of ROS by AGEs. Immunofluorescent staining indicated that AGEs markedly increased the expression of profilin-1 in the cytoplasm and the formation of actin stress fibres, resulting in the rearrangement and redistribution of the cytoskeleton. This effect was significantly ameliorated by DPI, GF 109203X, BAY-117082 or siRNA treatment of profilin-1. Incubation with DPI and GF 109203X markedly inhibited the activation of PKC triggered by AGEs, and DPI and BAY-117082 significantly decreased the activity of NF-κB mediated by AGEs. Disruption of profilin-1 gene expression attenuated the extent of endothelial abnormalities by reducing ICAM-1 and ADMA levels and elevating NO levels (P<0.05, P<0.01), but this disruption had no effect on the activities of NF-κB and PKC (P>0.05).

**Conclusions:**

These findings suggested that profilin-1 might act as an ultimate and common cellular effector in the process of metabolic memory (endothelial abnormalities) mediated by AGEs via the ROS/PKC or ROS/NF-қB signalling pathways.

## Background

Diabetes mellitus (DM) is a serious and rapidly growing disease, and diabetes-related vascular complications are major causes of patient disability and death. Large-scale clinical studies have confirmed that early intensive blood glucose control can reduce the incidence of diabetic microvascular and macrovascular complications. However, for patients with chronic long-term hyperglycaemia, despite strict future long-term glycaemic control, diabetes-related vascular complications remain or can develop. This phenomenon is known as “metabolic memory” or “hyperglycaemia memory.” There is growing evidence that faster-generated advanced glycation end products (AGEs) in the conditions of long-term high glucose may be a unifying explanation for this phenomenon [1]. Previous studies have demonstrated that AGEs are involved in the pathogenesis of endothelial dysfunction in diabetic vascular complications, and its levels in diabetic patients are highly correlated with the severity of macrovascular and microvascular complications [2,3]. However, the mechanism of “metabolic memory” mediated by AGEs has not been fully elucidated to date. The direct effects of AGEs include protein glycation and crosslinking, which affect normal protein physiological functions. In addition, this process is prolonged and irreversible. There is a growing body of evidence that AGEs mediate “metabolic memory”, primarily via indirect pathways via its receptors (receptor for advanced glycation end products, RAGE) [4]. Indeed, binding of AGEs to RAGE produces the excess formation of reactive oxidative species (ROS) independent of actual glucose, which subsequently activates protein kinase C (PKC) and the redox-sensitive transcription factor nuclear factor kappa B (NF-κB) via intracellular signalling cascade reactions. This activation subsequently initiates the expression of a variety of diabetes-related genes and RAGE [5]. Thus, self-maintaining conditions linked to AGE formation demonstrate that AGEs can conceivably contribute to “metabolic memory.”

Profilin-1 as an actin-binding protein is a class of small molecule proteins (12 to 15 KD) and is widely distributed in various types of cells with highly conserved sequences. This plays an important role in the regulation of actin polymerisation in a number of motility functions. The reorganisation and redistribution of the cytoskeleton, particularly actin proteins, forms a pathological basis for endothelial cell contraction and increased vascular permeability, which contributes to endothelial abnormalities and vascular disease [6]. Under pathological conditions, such as diabetes or atherosclerosis (AS), profilin-1 levels were increased in atherosclerotic lesions, the aorta or in serum. It was recently reported that profilin-1 overexpression triggered indicators of endothelial dysfunction and attenuated the expression of profilin-1 conferred protection from AS in vivo [7,8]. In addition, Romeo et al. reported that profilin-1 and low density lipoprotein (LDL) was downstream molecules mediating diabetic endothelial dysfunction, and revealed that the endothelial damage triggered by the profilin-1 pathway in diabetes and in lipid oxidation was surprisingly similar [7,9]. Thus, we hypothesized that profilin-1 may be a common and ultimate pathway in endothelial cell injuries, and blockade of profilin-1-mediated biological effects may help to prevent the occurrence of endothelial abnormalities and vascular disease.

Recent studies have demonstrated that incubation of endothelial cells with AGEs caused a significant increase in endothelial permeability via cytoskeletal alterations and actin rearrangement [10,11]. Due to the pivotal role of profilin-1 in the reorganisation and redistribution of actin, we propose that profilin-1 may be involved in metabolic memory mediated by AGEs as an ultimate pathway in endothelial injury. Thus, the present study aimed to elucidate the role of profilin-1 in endothelial injury mediated by AGEs and its underlying signal transduction pathways.

## Materials and methods

### Chemicals and reagents

Human umbilical vein endothelial cells (HUVEC12,ATCC,CRL-2480) were obtained from the Tumor Research Institute of Beijing Medical University (Beijing, China). Dulbecco’s modified Eagle’s medium (DMEM), Trizol reagents and Phallotoxins were obtained from Invitrogen. Foetal bovine serum (FBS) was supplied by Every Green Co. Ltd (Hangzhou, China). AGE-bovine serum albumin (AGE-BSA), diphenyliodonium (DPI), GF 109203X and BAY-117082 were purchase from Merck. Asymmetric dimethylarginine (ADMA) standard was purchased from Sigma. Intercellular adhesion molecule-1 (ICAM-1) ELISA kits and Griess reagents were purchased from Jiancheng Biological Medical Engineering Institute (Nanjing, China). ROS detection kits and BCA protein kits were purchased from Beyotime Company (Jiangsu, China). Rabbit anti-human-profilin-1 antibody was purchased from Santa Cruz. Western blotting kits and secondary rat anti-rabbit IgG were purchased from KPL. First strand cDNA synthesis kit was obtained from Fermentas. The primers were synthesised by Takara (Dalian, China). TRIzol, the PepTag Assay for Non-Radioactive Detection of Protein Kinase C and pGL4.32[luc2P/NF-κB-RE/Hygro] Vector was obtained from Promega. pLNCX2-siprofilin-1 plasmids were synthesised by Clonetech and lipofectamine 2000 was supplied by Invitrogen.

### Cell culture

HUVEC12 cells were maintained in DMEM containing 10% foetal bovine serum, 100 U/ml penicillin, and 100 μg/ml streptomycin and grown in humidified atmosphere of 5% CO_2_ in air at 37°C. HUVEC12 cells were cultured in six-well plates at a density of 5×10^4^ cells per well in DMEM until 70%-80% confluence. Each well was then washed twice with phosphate-buffered saline (PBS), and 2 ml of DMEM containing various concentrations of AGEs (100 μg/ml, 200 μg/ml, 400 μg/ml) were added for different time periods (0, 6, 12, 24, 48 h). To elucidate the potential signal pathways, DPI (antioxidants, 10 μmol/L), GF 109203X (PKC inhibitor, 10 μmol/L) and BAY-117082 (NF-κB inhibitor, 5 μmol/L) were used in the study.

### RNA isolation and real-time PCR analysis

Total RNA was extracted from cells grown in a 6-well plate using Trizol reagent and cDNA was synthesised from 1 μg total RNA using the First-Strand Synthesis System for PCR according to the manufacturer’s protocol. The primer pairs used in amplification of profilin-1 were: forward primer, 5′- CTGTCAGGACGCGGCCATCG -3′; reverse primer, 5′-CAGCTGGCGTGATGTTGACGA-3′. The primer pairs of GAPDH were: forward primer, 5′-GTCGCCAGCCGAGCCACATC-3′; reverse primer, 5′-CCAG GCGCCCAATACGACCA-3′. The cDNA was amplified using the SYBR PCR Master mix and 0.4 μL of each primer pair. The amplification was performed with an initial step at 95°C for 10 min, and 40 cycles of denaturation at 95°C for 15 s, annealing at 60°C for 15 s and extension at 72°C for 15 s for profilin-1 and GAPDH. Under optimised conditions, there was a single melting curve and no primer-dimer formation. The copy number for each mRNA was determined using a standard curve generated with external standards of a known copy number. All amplification reactions for each sample were performed in triplicate and the results were expressed as the ratio of profilin-1 to GAPDH mRNA.

### Protein preparation and western blotting analysis

After treatment, the cells were lysed and the protein concentrations were measured using the BCA protein assay. The supernatants were separated using 5% SDS-PAGE for 1.5 h and transferred onto a polyvinylidene difluoride (PVDF) membrane followed by 2 h incubation in 5% non-fat milk in PBST (0.1% Tween 20 in PBS) or at 4°C overnight. The blot was probed using an antibody against rabbit anti-human-profilin-1 (1:1500) and subsequently incubated with FITC-conjugated secondary rat anti-rabbit IgG protected from light. The signal was detected and ratios of the target protein against β-actin control were calculated using the Odyssey Infrared Imaging System (LI-COR Biosciences).

### Immunofluorescent staining

The formation of actin stress fibres as an index of endothelial cytoskeletal reorganisation was analysed using fluorescence microscopy. Endothelial cells on coverslip were fixed in 3.7% formaldehyde for 10 min at room temperature and extracted with 0.1% Triton X-100 for 5 min. Each coverslip was incubated with a rabbit anti-human antibody against profilin-1 (1:50) and then incubated with a FITC-conjugated secondary rat anti-rabbit IgG (imaged on the green channel). F-actin in the cytoplasm was stained with phallotoxins (1:200) (imaged on the red channel) in 1% BSA in TBST for 1 h. The cells were then rinsed four times with PBS to remove excess antibodies, and then labelled with 1% 4′,6-diamidino-2-phenylindole (DAPI) in PBS for 5 min to visualise nuclei. Non-immune rabbit IgG and no phallotoxins were used as a negative control in consecutive sections. Microscopy was performed using the IX81 FV1000 laser confocal scanning microscope (Olympus, Japan).

### Supernatant ICAM-1, NO, and ADMA detection

ICAM-1 in supernatants were detected using an ELISA kit. The level of nitric oxide (NO) in the medium was determined indirectly by the content of nitrite and nitrate using the Griess reagent, and the absorbance was determined at 540 nm using a spectrophotometer. The supernatants were obtained for the measurement of ADMA content using high-performance liquid chromatography (HPLC).

### Intracellular ROS detecting

After the cells of each group were treated, the cell culture medium was removed, and 1 ml 2′,7′-dichlorofluorescin diacetate (DCFH-DA) (1:500 diluted concentration) DMEM was added to cover the cells completely. The cells were cultured for an additional 30 min. After rinsing with serum-free DMEM three times to remove free DCFH-DA, the level of ROS in the HUVEC12 cells was observed using the IX81 FV1000 laser confocal scanning microscope (Olympus, Japan) with an excitation wavelength at 488 nm.

### Measurement of PKC activity

Endothelial cells (5×10^6^ to 1×10^7^) were extracted according to the manufacturer’s protocol. The amount of phosphorylated and nonphosphorylated peptide species was detected using spectrophotometry at a length of 570 nm. The kinase activity was quantified using the PepTag Assay for Non-Radioactive Detection of Protein Kinase C.

### Measurement of NF-κB activity

NF-κB activity in endothelial cells was detected using an NF-κB-luciferase reporter vector (pGL4.32[luc2P/NF-κB-RE/Hygro] vector). Briefly, 5×10^6^ endothelial cells were transiently co-transfected with pSV-β-galactosidase and pGL4.32[luc2P/NF-κB-RE/Hygro] using liposome-mediated transfection at a DNA/lipid ratio of 1 μg of each plasmid DNA/2 μl of Lipofectamine 2000. The cells were allowed to recover for 24 h prior to being plated into 24-well plates at 1×10^5^ cells/well and the luciferase activity was measured using a Victor 4 multi-labelled counter and the Luciferase Assay System kit according to manufacturer’s instructions. The intensity of the luciferase activity in these cells was normalised against the β-galactosidase activity, which was used as an internal control. The luciferase activity was expressed as a fold increase.

### RNA interference and cell transfection

To silence profilin-1 gene expression, pLNCX2-siprofilin-1 plasmids were transfected into HUVEC12 cells using Lipofectamine 2000 when the cells were 70% confluent according to the manufacturer’s protocol. The transfection efficiency for each experiment was determined using the percentage of the cells that expressed GFP (green fluorescent protein) under a fluorescent microscope (Nikon, Japan) or by western blotting analysis and real-time PCR.

### Statistical analysis

All data were expressed as the mean ± SD. The ANOVA test was used to detect differences between groups and the LSD or Dunnett T3 test was used for multiple comparisons. A value of P<0.05 was considered significant.

## Results

### The mRNA and protein expression of profilin-1 in endothelial cells

**Figure 1:**
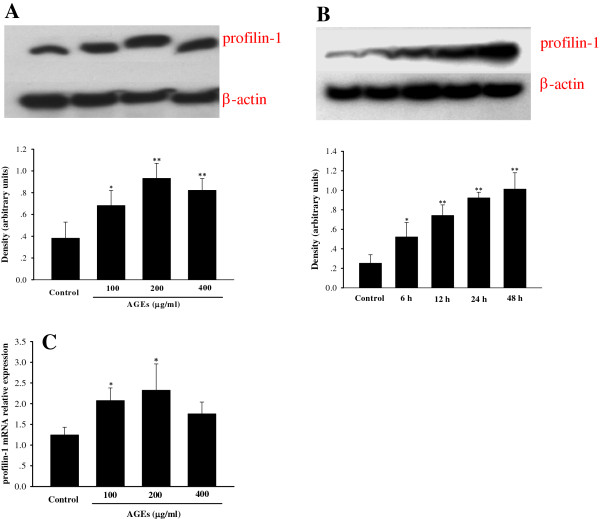
**Effect of AGEs on the expression of profilin-1 in HUVEC12 cells. A** (dose study): AGEs (100 μg/ml), AGEs (200 μg/ml), AGEs (400 μg/ml): Endothelial cells were incubated with AGEs (100 μg/ml, 200 μg/ml, 400 μg/ml) for 24 h. **B** (time study): 6 h, 12 h, 24 h, 48 h: endothelial cells were incubated with 200 μg/ml AGEs for 6 h, 12 h, 24 h, 48 h. **C**: Effect of AGEs on the mRNA expression of profilin-1: Endothelial cells were incubated with AGEs (100 μg/ml, 200 μg/ml, 400 μg/ml) for 4 h. n=3, ^*^P < 0.05 vs control, ^**^P < 0.01 vs control.

**Figure 2:**
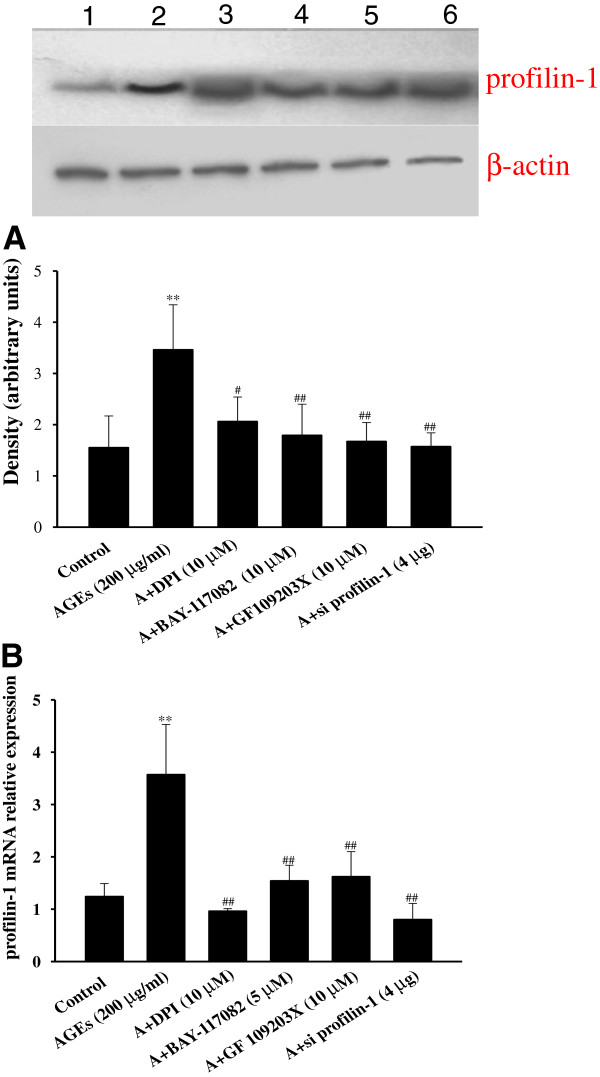
**Effect of different inhibitors on the expression of profilin-1 induced by AGEs in endothelial cells. A**: the protein expression of profilin-1 by western blotting; **B**: the mRNA expression of profilin-1 by real-time PCR. 1: control; 2: AGEs (200 μg/ml): endothelial cells were incubated with 200 μg/ml AGEs for 24 h; 3: A+DPI (10 μM): endothelial cells were incubated with 10 μmol/L DPI for 1 h prior to AGEs (200 μg/ml, 24 h); 4: A+BAY-117802 (5 μM): endothelial cells were incubated with 5 μmol/L BAY-117802 for 1 h prior to AGEs (200 μg/ml, 24 h); 5: A+GF 109203X (10 μM): endothelial cells were incubated with 10 μmol/L GF 109203X for 1 h prior to AGEs (200 μg/ml, 24 h); 6: A+si profilin-1 (4 μg): endothelial cells were incubated with 200 μg/ml AGEs and 4 μg pLNCX2-si profilin-1 for 4 h and then with 200 μg/ml AGEs for 20 h; n=3, ^**^P < 0.01 vs control, ^#^P < 0.05 vs AGEs (200 μg/ml), ^##^P < 0.01 vs AGEs (200 μg/ml).

Compared with the control, incubation of endothelial cells with AGEs (100 μg/ml, 200 μg/ml, 400 μg/ml) significantly up-regulated the mRNA and protein expression of profilin-1 at 4 h or 24 h (*P*<0.05, *P*<0.01). In addition, treatment with AGEs at a dose of 200 μg/ml had a most robust effect on profilin-1 gene and protein expression (Figure 1A, C), and demonstrated up-regulation of profilin-1 protein expression in a time-dependent manner (*P*<0.05, *P*<0.01, Figure 1B). Thus, AGEs at a dose of 200 μg/ml for 24 h was selected for further studies. Pretreatment with DPI (10 μmol/L), GF 109203X (10 μmol/L) and BAY-117082 (5 μmol/L) significantly decreased the up-regulated mRNA and protein expression of profilin-1 mediated by AGEs (200 μg/ml, 24 h) (Figure 2).

### Endothelial cells abnormalities

**Figure 3:**
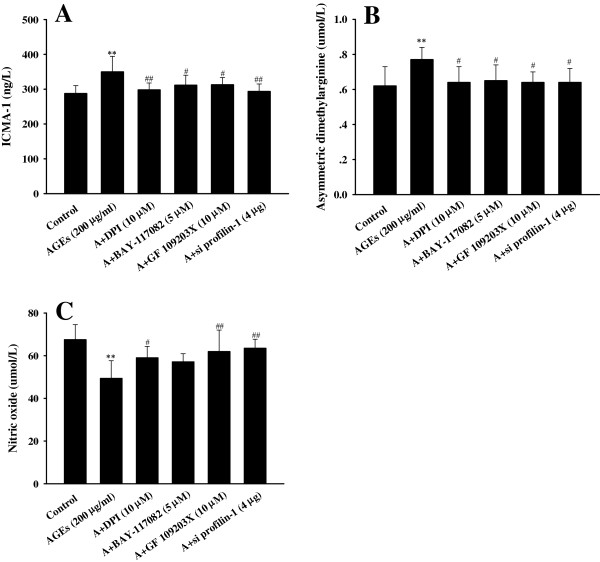
**Effect of different inhibitors on the levels of cytokines induced by AGEs in endothelial cells. A**: the levels of ICAM-1; **B**: the levels of ADMA; **C**: the levels of NO. n=6, ^**^P < 0.01 vs control, ^#^P < 0.05 vs AGEs (200 μg/ml), ^##^P < 0.01 vs AGEs (200 μg/ml).

In cultured endothelial cells, treatment with AGEs (200 μg/ml, 24 h) markedly increased the levels of ICAM-1 and ADMA, and decreased the synthesis of NO, which was attenuated by pretreatment with DPI, GF 109203X and BAY-117082 (*P*<0.05, *P*<0.01, Figure 3).

**Figure 4:**
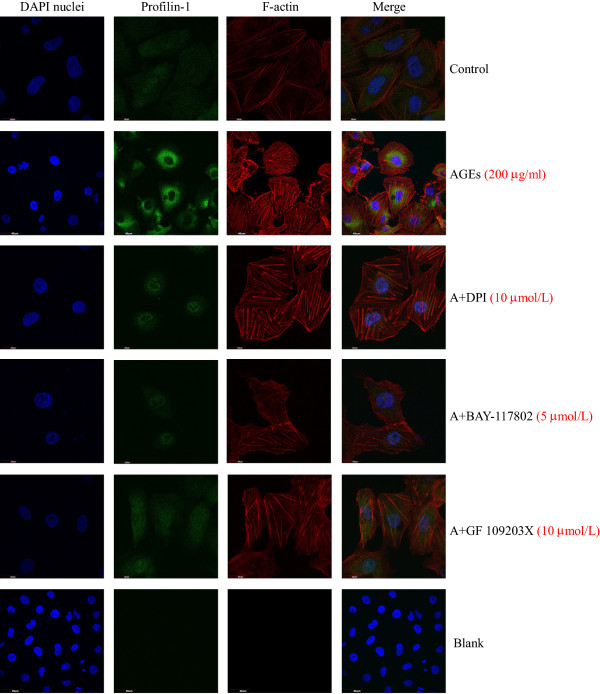
**Effect of different inhibitors on the expression of profilin-1 and the distribution of F-actin induced by AGEs in endothelial cells.** n=3. Immunofluorescence staining for profilin-1 (green), F-actin (red) and 4′,6-diamidino-2-phenylindole (DAPI) nuclei (blue). Non-immune rabbit IgG was used as blank.

Actin stress fibre formation in endothelial cells during stress is a commonly recognised cytoskeletal response, which indicates reorganisation of the actin cytoskeleton [12]. Thus, we assessed the effects of AGEs on actin cytoskeletal redistribution using fluorescence confocal microscopy, which enables visualisation of profilin-1 and actin filaments. As shown in Figure 4, F-actin was well-distributed at the edge of the cytomembrane in the control group; however, F-actin fibre morphology and distribution showed a marked change after treatment with AGEs. AGEs (200 μg/ml, 24 h) induced the appearance of many coarse and short actin stress fibres in the cytoplasm. Immunofluorescent staining showed that the level of green fluorescence in the AGE treatment group was significantly higher than the control group, further supporting that the expression levels of profilin-1 were up-regulated with AGE treatment. Pretreatment with DPI, GF 109203X and BAY-117082 markedly lowered the green fluorescent expression and decreased the formation of actin stress fibre in the cytoplasm.

### The protective role of profilin-1 siRNA in AGE-induced endothelial damage

**Figure 5:**
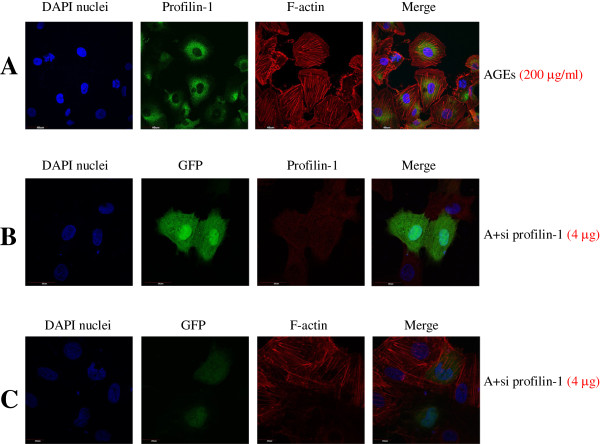
**Effect of profilin-1 siRNA on the expression of profilin-1 and the distribution of F-actin induced by AGEs in endothelial cells.** n=3. **A**: AGEs: endothelial cells were incubated with 200 μg/ml AGEs for 24 h. Immunofluorescence staining for profilin-1 (green), F-actin (red) and DAPI nuclei (blue). **B**: A+si profilin-1 (4 μg): endothelial cells were incubated with 200 μg/ml AGEs and 4 μg pLNCX2-si profilin-1 for 4 h and then with 200 μg/ml AGEs for 20 h. Immunofluorescence staining for green fluorescent protein (GFP) (green), profilin-1 (red) and DAPI nuclei (blue). **C**: A+si profilin-1 (4 μg): Immunofluorescence staining for GFP (green), F-actin (red) and DAPI nuclei (blue).

To confirm the role of profilin-1 in AGE-mediated endothelial injury, we generated specific pLNCX2-siprofilin-1 plasmids to knock down the expression of profilin-1. Pretreatment with 4 μg pLNCX2-siprofilin-1 plasmids successfully knocked down the mRNA and protein expression of profilin-1 induced by AGEs in HUVECs (*P*<0.01, Figure 2). Importantly, profilin-1 siRNA blunted the endothelial injury induced by AGEs (200 μg/ml, 24 h) as shown by the significant decrease in the levels of ICAM-1 and ADMA, and the increase in NO production (P<0.05, P<0.01, Figure 3). Immunofluorescent staining showed that silencing profilin-1 gene expression markedly decreased the expression of profilin-1 (red fluorescence, Figure 5B) in the cytoplasm and improved F-actin redistribution (red fluorescence) in the presence of AGEs (Figure 5C). Overall, these results confirmed that AGEs induced actin cytoskeletal reorganisation and redistribution via increasing the levels of profilin-1 in the cytoplasm.

### Involvement of PKC and the NF-κB pathway in AGE-induced endothelial injury

**Figure 6:**
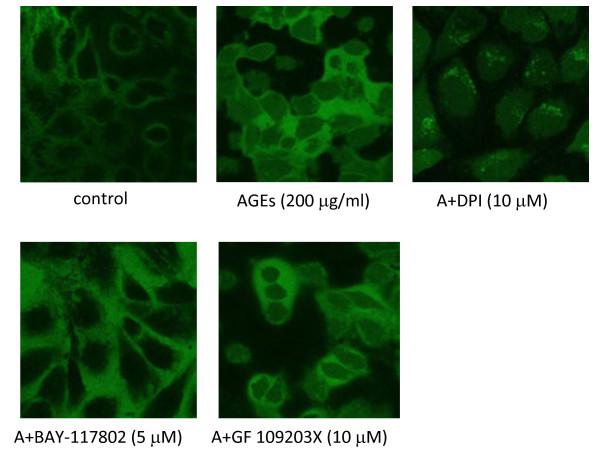
**Effect of different inhibitors on the levels of intracellular ROS mediated by AGEs in endothelial cells.** n=3.

**Figure 7:**
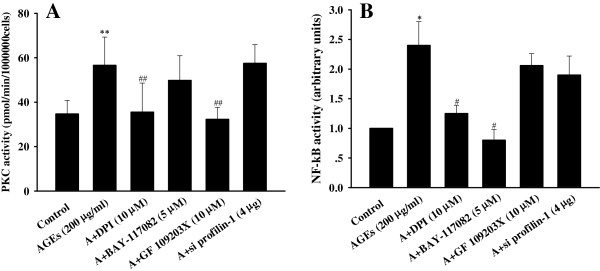
**Effect of different inhibitors on the signaling pathways mediated by AGEs in endothelial cells. A**: the activity of PKC; **B**: the activity of NF-қB. n=4, ^*^P < 0.05 vs control, ^**^P < 0.01 vs control, ^#^P < 0.05 vs AGEs (200 μg/ml), ^##^P < 0.01 vs AGEs (200 μg/ml).

To explore the potential signal pathway involved in AGE-induced endothelial injury, DPI (antioxidant), GF 109203X (PKC inhibitor) and BAY 117082 (NF-κB inhibitor) were incubated for 1 h prior to AGEs stimulation. These results showed that antioxidants or blockade of the PKC or NF-κB pathways significantly improved the endothelial abnormalities induced by AGEs, concomitantly with down-regulation of profilin-1 expression (Figures 2, 3, 4). In addition, DPI markedly decreased the elevated levels of ROS induced by AGEs (200 μg/ml, 24 h); however, GF 109203X and BAY-117082 showed no significant effect on ROS formation induced by AGEs (Figure 6). Because marked green fluorescence showed mutual interference, the intracellular ROS levels were not determined in the interference study. Expectedly, DPI and GF 109203X significantly inhibited the activity of PKC, and DPI and BAY-117082 markedly prevented the activation of NF-κB (*P*<0.05, *P*<0.01). However, GF 109203X had no effect on the activity of NF-κB, and BAY-117082 had no effect on the activity of PKC (*P*>0.05). Blockade of profilin-1 expression also had no effect on the activation of PKC and NF-κB induced by AGEs (Figure 7). These results suggested that AGEs mediated endothelial abnormalities via the excess formation of ROS and subsequent activation of the PKC and NF-κB pathways. Furthermore, profilin-1 is the ultimate and common downstream effector in endothelial injury.

## Discussion

The main findings of the present study are as follows: (1) AGEs induced endothelial injury as shown by increasing ADMA and ICAM levels and decreasing the synthesis of NO, which occured concomitantly with the up-regulated expression of profilin-1 and the rearrangement and redistribution of F-actin. This was attenuated by treatment with the antioxidant DPI, PKC inhibitor GF109203X or NF-κB inhibitor BAY117082; (2) Knockdown of profilin-1 gene expression attenuated AGE-induced endothelial abnormalities; (3) AGEs up-regulated the expression of profilin-1 via the excess production of ROS and subsequent activation of PKC and NF-κB. Taken together, these findings suggested, for the first time, that profilin-1 plays an important role in endothelial injury induced by AGEs, which may contribute to macrovascular complications in DM.

There is accumulating evidence that AGEs formation due to chronic hyperglycaemia has a chemical, cellular and tissue effect in metabolic memory. AGEs have been previously associated with the development of diabetes-related macrovascular and microvascular complications [13-15]. It has been recently reported that dietary AGEs in diabetic patients or intraperitoneal injection of AGEs in rats caused an impairment in the vascular endothelium [16-18], which was prevented by treatment with the AGE inhibitor benfotiamine [16]. Accumulation of AGEs in the vasculature initiated a series of morphological and functional changes in endothelial cells and induced an increase in endothelial permeability and cell apoptosis. Moreover, it also promoted endothelial (progenitor) cell migration, adhesion and focal contact formation, concomitantly with the decreased synthesis of NO and the activity of superoxide dismutase (SOD) [10,18,19]. It is well-known that the vascular endothelium is an important target of hyperglycaemic damage and an increase in endothelial permeability to macromolecules results in vascular dysfunction that is associated with several pathological states, including diabetes. Previous studies have demonstrated that the elevated ADMA levels connected with the uncoupling of NO synthesis contributing to endothelial dysfunction were associated with future cardiovascular events in diabetic patients [20]. Indeed, ADMA has been recognised as a marker of endothelial dysfunction and as a risk factor of cardiovascular diseases [21,22]. It was recently reported that AGEs markedly increased ADMA levels in tubular and endothelial cells via the stimulation of local ROS production [23,24]. A great deal of studies have demonstrated that circulating level of sICAM-1 is considered to be one important marker of endothelial dysfunction and it was reported that the endothelial dysfunction marked by impaired acetylcholine-induced endothelium-dependent relaxation of aortic rings and elevated levels of sICAM-1 were present in streptozocin-induced diabetic rats [25]. In the present study, we explored the effect of AGEs on the adverse actions in cultured endothelial cells. Consistent with previous studies, incubation with AGEs markedly reduced the synthesis of NO, increased the levels of ADMA, ICAM-1 and intracellular ROS, which was attenuated by pretreatment with the antioxidant DPI, suggesting that exogenous AGEs directly induced endothelial injury via the overproduction of ROS.

Profilin-1, an intracellular actin-binding protein, achieves its function via the regulation of the size, localization and dynamics of unpolymerised actin in cells contributing to endothelial cell contraction and vascular hyperpermeability. Previous studies have demonstrated that cardiovascular risk factors, such as homocysteine, LDL, ox-LDL, and oxidised cholesterol can up-regulated the expression of profilin-1 in cultured endothelial cells, resulting in cytoskeletal structural remodelling [8,26,27]. Recently, it has been reported that overexpression of profilin-1 up-regulated the expression of ICAM-1, increased endothelial cell permeability, induced endothelial cell apoptosis and promoted endothelial cell migration, adhesion and focal contact formation [7,9] and silencing profilin-1 gene expression provided significant protection on endothelial cells [28]. In vivo studies have further demonstrated that profilin-1 expression was up-regulated in endothelial cells and macrophages in atherosclerotic lesions in ApoE null mice, and in the aorta of diabetic rats, and the serum levels of profilin-1 were significantly elevated in patients with severe AS [8,29]. In contrast, attenuated expression of profilin-1 conferred protection against AS in LDL receptor null mice [8]. Recently, Romeo et al. reported that high-fat diet (HFD) up-regulated the expression of profilin-1 in both stromal vascular cells and adipocytes of white adipose tissue (WAT) and pfn heterozygote mice (PfnHet) displayed near normalization of HFD-induced glucose intolerance and the release of pro-inflammatory cytokines, suggesting that profilin-1 plays an bran-new role in modulation of immune homeostasis within the WAT microenvironment [30]. Because endothelial damage stimulated by profilin-1 in diabetes is surprisingly similar with that in lipid oxidation, it is conceivable that profilin-1 may function in a common pathway that induces vascular endothelial injuries in vascular lesions. It has been previously reported that incubation with AGEs caused a significant increase in the permeability of endothelial cell monolayers via significant disorganisation of the F-actin cytoskeleton and disruption of tight and adherent junctions [10,11,31-33]. However, detailed studies on the role of profilin-1 in AGE-induced adverse actions in vascular endothelial cells are lacking. In addition, the protection offered by specifically blocking the expression of profilin-1 against AGE-induced endothelial alterations has not yet been reported. This study found that treatment with AGEs markedly up-regulated the mRNA and protein expression of profilin-1, which was accompanied by the rearrangement and redistribution of the cytoskeleton. However, silencing profilin-1 expression significantly attenuated endothelial injury by increasing the synthesis of NO and decreasing the levels of ADMA and ICAM-1. This occurred concomitantly with the attenuation of the cytoskeletal rearrangement. To the best of our knowledge, this is the first report demonstrating that AGEs up-regulated the expression of profilin-1 in vascular endothelial cells and the silencing of profilin-1 expression induced endothelial protection against AGEs effects.

It is well known that AGEs mediate metabolic memory via its receptor RAGE to produce excess ROS, and subsequently activates PKC and NF-κB signalling via intracellular signalling cascades. This process induces the expression of a variety of diabetes-related genes [1,5]. Normalisation of the levels of mitochondrial ROS prevented glucose-induced activation of PKC and NF-κB, the formation of AGEs and sorbitol accumulation [34]. It has been previously reported that AGEs induced PKC-β translocation, extracellular signal-regulated protein kinase 1/2 and NF-κB activation in bovine retinal endothelial cell, and pharmacological inhibition of these signalling pathways and antioxidants abolished the effects mediated by AGEs [35]. Substantial evidence indicates that activation of PKC and NF-κB is a key biochemical event implicated in the development of diabetic vascular complications [36,37]. To elucidate the potential signal pathway involved in endothelial injury induced by AGEs, antioxidant DPI, PKC inhibitor (GF 109203X) and NF-κB inhibitor (BAY-117082) were employed. These results showed that pretreatment with DPI, GF 109203X and BAY-117082 attenuated endothelial injury, down-regulated the elevated expression of profilin-1 and ameliorated the actin cytoskeleton rearrangement and redistribution induced by AGEs. Antioxidant DPI markedly inhibited the formation of ROS and activation of PKC and NF-κB signalling by AGEs. However, blockade of the PKC and NF-κB pathways had no significant effect on the overproduction of ROS. It was previously reported that shear stress increased the production of ROS and the activity of PKC in aortic endothelial cells, and the increase in ROS production was unaffected by GF109203X, whereas the activation of PKC was reduced by antioxidant N-acetyl-L-cysteine (NAC) [38]. Thus we concluded that oxidative stress triggered by AGEs subsequently activated downstream NF-κB and PKC signalling pathways. Interestingly, silencing of profilin-1 gene expression induced endothelial protection, but did not affect the activity of NF-κB and PKC. Thus, it can be inferred that AGEs up-regulated the expression of profilin-1 and caused damage in endothelial cells via the production of excess ROS, thereby activating NF-κB and PKC signalling pathways. Thus, we speculated that profilin-1 functions as an ultimate and common cellular channel in endothelial abnormalities mediated by AGEs.

In conclusion, the present study suggests, for the first time, that profilin-1 is a downstream molecule that mediates endothelial injury induced by AGEs via the ROS/PKC or ROS/NF-κB signalling pathways, and blockade of profilin-1-mediated biological effects may help to prevent endothelial injuries and vascular lesions in diabetes. Taken together, these findings set the stage for a prominent role of profilin-1 as a modulator of the actin cytoskeleton, which may underlie the pathology of vascular diseases, including diabetes.

## Abbreviations

ADMA: Asymmetric dimethylarginine; AGE: Advanced glycation end products; AGE-BSA: AGE-bovine serum albumin; AS: Atherosclerosis; DAPI: 4′,6-diamidino-2-phenylindole; DCFH-DA: 2′,7′-dichlorofluorescin diacetate; DM: Diabetes mellitus; DMEM: Dulbecco’s modified eagle’s medium; DPI: Diphenyliodonium; FBS: Foetal bovine serum; GFP: Green fluorescent protein; HFD: High-fat diet; HPLC: High-performance liquid chromatography; HUVEC: Human umbilical vein endothelial cells; ICAM-1: Intercellular adhesion molecule-1; LDL: Low density lipoprotein; NAC: N-acetyl-L-cysteine; NF-κB: Nuclear factor kappa B; NO: Nitric oxide; PBS: Phosphate-buffered saline; PfnHet: Pfn heterozygote mice; PKC: Protein kinase C; PVDF: Polyvinylidene difluoride; RAGE: Receptor for advanced glycation end products; ROS: Reactive oxidative species; SOD: Superoxide dismutase; WAT: White adipose tissue.

## Competing interests

The authors declare that they have no competing interests.

## Authors’ contributions

MC conceived the study, arranged the collaboration, initiated the manuscript, edited and compiled the final version for submission. ZL and QZ performed laboratory work and data analysis. TY and XX participated in its design and coordination. All authors read and approved the final manuscript.
